# Self-employed and stressed out? The impact of stress and stress management on entrepreneurs’ mental health and performance

**DOI:** 10.3389/fpsyg.2024.1365489

**Published:** 2024-04-04

**Authors:** Sophia Kiefl, Sophie Fischer, Jan Schmitt

**Affiliations:** ^1^Faculty of Health Psychology, SRH Distance Learning University, Riedlingen, Germany; ^2^Institute of Digital Engineering, Technical University of Applied Sciences Würzburg-Schweinfurt, Schweinfurt, Germany

**Keywords:** stress, stress management, coping, mental health, self-employment, entrepreneurship

## Abstract

**Introduction:**

Entrepreneurs play a central role in economic and social stability, yet the start-up rate in Germany has declined in recent years, possibly due to the stress associated with entrepreneurial endeavors. Stressors such as financial uncertainty and time pressure are prevalent among entrepreneurs and negatively affect their psychological well-being. However, research on stress management strategies among self-employed individuals remains limited.

**Methods:**

This pilot study conducted a quantitative analysis with 117 self-employed participants in Germany. The study focused on typical entrepreneurial work demands and selected stress coping mechanisms.

**Results:**

The analysis revealed a significant correlation between quantitative demands and mental exhaustion. Furthermore, a high positive correlation between presenteeism and workload suggests that presenteeism may partially explain the variance in workload. These findings underscore how high job demands can lead to self-endangering behaviors that are detrimental to mental health.

**Discussion:**

Although no significant moderating effect of proactive coping on the relationship between job demands and mental exhaustion was observed, significant negative correlations between proactive coping and both job demands and mental exhaustion suggest a potential protective role of proactive coping against work-related stress. This study highlights the importance of understanding stress coping strategies among self-employed individuals and their impact on entrepreneurial success and mental well-being. Further research in this area is warranted to develop effective interventions to support the well-being and productivity of self-employed individuals in Germany.

## 1 Introduction

Numerous studies emphasize entrepreneurs’ significant contribution to economic growth and social stability (Stoica et al., 2020; Fischer et al., 2021; Gomes et al., 2023). According to Spinelli and Adams (2014), entrepreneurs are characterized as experienced strategists with a broad mindset, focusing their decision-making processes on the implementation of visions and concepts. Entrepreneurship entails recognizing opportunities and possessing the determination, adaptability, and risk-taking capacity to create value and promote social progress (Spinelli and Adams, 2014; Fischer et al., 2021). In Germany, federal and state governments respond with funding programs aimed at providing targeted support for company foundations and startups (Fischer et al., 2021). Additionally, the number of private-sector support facilities, such as incubator and accelerator programs, co-working spaces, and maker labs, is increasing (Zinke et al., 2018, pp. 33–95; Fischer et al., 2021). However, despite the numerous support services, the startup rate in Germany has been steadily declining since 2012 (Bonin et al., 2022, p. 16; Metzger, 2022, p. 1). In the first quarter of 2023, the number of self-employed individuals in Germany was approximately 3.9 million compared to 41.7 million employees, representing around 8.5% of all employees in Germany. This reflects a decrease of 0.8% in the number of self-employed individuals compared to the previous year (Federal Statistical Office, 2023b). One possible explanation for the low proportion of entrepreneurs in Germany is the association of entrepreneurial activity with high levels of professional stress (Kieschke and Schaarschmidt, 2003, p. 108; Stephan, 2018, p. 290).

Entrepreneurs are often exposed to higher levels of stress compared to other occupational groups, which leads to negative health effects (White and Gupta, 2020; Lee et al., 2023; Wang et al., 2023, p. 81). The work demands faced by the self-employed have been extensively studied in the literature in terms of stress levels (White and Gupta, 2020; Lee et al., 2023; Wang et al., 2023). Effectively managing the development and management of stress in the self-employed is therefore a critical success factor that impacts not only the health of the entrepreneur, but also the performance and prosperity of the business (Wach et al., 2021). In recent years, researchers have increasingly focused on the specific stressors faced by the self-employed (Wach et al., 2021; Lee et al., 2023; Wang et al., 2023). Wach et al. (2021) emphasize that the well-being of entrepreneurs is increasingly taking center stage as it directly correlates with business performance and serves as a measure of entrepreneurial success. Therefore, it is imperative to explore strategies that enable entrepreneurs to maintain or enhance their well-being during a demanding workload (Wach et al., 2021; Wang et al., 2023).

High financial and economic uncertainty as well as considerable time and deadline pressure are often cited in the literature as primary stressors in entrepreneurial ventures (Kottwitz et al., 2019; Lek et al., 2020; White and Gupta, 2020). Especially in times of financial uncertainty, entrepreneurs are confronted with increased expectations of flexibility, which can have a negative impact on their mental well-being. For example, they must respond flexibly to orders to ensure financial stability, even if their time resources are limited (Kottwitz et al., 2019). Entrepreneurs must also respond skillfully to rapid economic fluctuations (Fischer et al., 2021). In addition, high workload is repeatedly mentioned in the literature as a stressful aspect of the entrepreneurial environment, with workload meaning an overwhelming amount of tasks, often accompanied by long working hours, which has a negative impact on mental health (Stephan, 2018; Wach et al., 2021).

Stress can significantly affect job satisfaction, performance and commitment to the organization (Eager et al., 2015, p. 254; Wach et al., 2021). The mental health of entrepreneurs is a critical factor for the sustainable preservation of companies (Eager et al., 2015, p. 254; Lee et al., 2023). Against this backdrop, increasing attention is being paid to the stress management skills of entrepreneurs. The way in which entrepreneurs deal with stressful working conditions can be crucial for maintaining their health and thus for the sustainable success of the company. Conversely, maladaptive coping mechanisms can jeopardize the health of entrepreneurs and thus the company (Kieschke and Schaarschmidt, 2003, p. 109; Neck et al., 2013, p. 475). Therefore, this study aims to investigate the relationship between workloads, coping strategies and the mental well-being of self-employed workers. The research is guided by the following questions:

*RQ1*: To what extent does work stress affect the mental health of the self-employed?

*RQ2*: To what extent do health-endangering coping strategies affect the relationship between workload and well-being?

*RQ3*: To what extent do health-promoting strategies affect the relationship between workload and well-being?

This paper presents the results of a quantitative pilot study conducted in the context of an online network with 117 participants. The sample represents a simple, non-probabilistic self-selection designed to reflect the diversity of the self-employed in Germany. Our study focuses on typical entrepreneurial work demands, including cognitive and quantitative aspects, as well as selected stress coping strategies such as work extension, presenteeism, proactive coping and reframing, and examines their influence on the mental exhaustion experienced by the self-employed respondents. While our assessment is not exhaustive, it provides valuable insights into the use of self-threat coping strategies, proactive coping and emotional coping through positive reframing. The study therefore contributes to the entrepreneurship literature by examining self-employed individuals’ stress coping mechanisms and highlighting how quantitative demands play a significant role in mental exhaustion, as well as how presenteeism mediates work stress. Furthermore, we emphasize the importance of understanding stress coping strategies for entrepreneurial success and psychological well-being within the current entrepreneurship research landscape (e.g., Lee et al., 2023; Wang et al., 2023).

The article is divided into five sections. After the introduction (Section 1), Section 2 (Theoretical background) explains the empirical studies and theoretical models on which our hypotheses are based. Section 3 (Methods) describes the detailed research design, including a description of the sample and the analytical approach. The results of our hypothesis testing are described in Section 4 (Results) and then discussed and contrasted with the underlying theories in Section 4 (Discussion). Finally, Section 5 (Conclusion) summarizes the results and limitations of our study, while highlighting avenues for future research.

## 2 Theoretical framework

### 2.1 Job demand-resources model

The Job Demand-Resources (JD-R) according to Bakker and Demerouti (2017) is a model to explain the development of burnout or mental exhaustion and work. Based on the stress–strain model and work design theories (Demerouti and Nachreiner, 2019; Wang et al., 2023), the JD-R model integrates these theoretical frameworks. The stress–strain model describes the interplay between work demands (stressors) and stress reactions (strain), while work design theories clarify the relationship between work resources and work motivation. By bringing these perspectives together, the JD-R model comprehensively clarifies the effects and relationships of work demands and resources on burnout and work engagement (Demerouti and Nachreiner, 2019, p. 120; Wang et al., 2023). The model is based on two fundamental premises: First, burnout risk factors can be categorized into two main groups: job demands and job resources. Second, these categories, work demands and work resources, can trigger two different psychological mechanisms.

The authors define work demands as occupational activities that are typically associated with persistent physical and/or psychological stress and specific physiological and/or psychological costs caused by physical, psychological, social and organizational factors (Demerouti and Nachreiner, 2019, p. 121). Examples include time pressure or high cognitive demands (Demerouti and Nachreiner, 2019, p. 121). Work demands have been identified as the most important predictors of mental exhaustion (Bakker and Demerouti, 2017, p. 274). Work resources are understood as all physical, psychological, social and organizational factors that support a person in achieving their goals, dealing appropriately with work demands and stressors and serving personal development (Schuster et al., 2011, p. 51; Demerouti and Nachreiner, 2019, p. 121).

In the first process, constantly high work demands lead to mental exhaustion in the long term (Demerouti and Nachreiner, 2019, p. 122). In the second process, a lack of work resources leads to frustration and subsequently to a decline in motivation and work commitment (Bakker et al., 2004, pp. 87–88; Demerouti and Nachreiner, 2019, p. 122). The effect of these two processes has been empirically confirmed in several studies (Bakker and Demerouti, 2007, p. 317). In addition, a third effect has been identified and empirically confirmed: Work resources fulfill a buffering effect between work demands and mental exhaustion (Bakker et al., 2004, pp. 88–98; Bakker and Demerouti, 2007, p. 314; Demerouti and Nachreiner, 2019, p. 124). The model can be represented graphically as follows:

Wrzesniewski and Dutton (2001) and Xanthopoulou et al. (2007) have considerably extended the JD-R model by including personal resources and job crafting. Xanthopoulou et al. (2007) included personal resources such as self-efficacy, organizational self-esteem and optimism in the model and examined their moderating and mediating roles. Although the moderating effect could not be confirmed, personal factors were found to mediate between work resources and work engagement. In particular, personal resources were found to play an active-preventive role by activating personal resources to reduce mental exhaustion. This underlines the importance of personal resources alongside work demands and resources in the JD-R model. Job crafting, as proposed by Wrzesniewski and Dutton (2001), represents a proactive, self-initiated perspective on changing working conditions. It involves various changes that individuals make to their tasks, cognitive attitudes and working relationships. Job crafting is relevant to this study as it suggests coping strategies such as proactive coping and reframing of situations as intrapsychic resources that mitigate the detrimental effects of high work demands on mental exhaustion.

For this study, the JD-R model is of central importance to the empirical investigation. With its valid empirical foundations and extensions, in particular job crafting, it offers insights into the interaction between resource-oriented coping strategies, job demands and mental health.

### 2.2 Stress levels among self-employed persons

The International Classification of Occupations (ISCO) is a globally recognized standard for categorizing types of employment (Schüller and Wingerter, 2019). The ISCO divides the self-employed into different groups: those with employees, such as managing proprietors and sole proprietors with staff; those without employees, also known as solo self-employed, which includes managing proprietors and sole proprietors without staff; and dependent self-employed, who work in their own business to generate profits but are heavily dependent on a commercial contractor. Furthermore, the distinction between consumer and entrepreneur is crucial and is regulated by the German Civil Code, which defines an entrepreneur as a natural or legal person who acts independently in the exercise of his or her trade, business or profession, with no distinction between self-employed persons and entrepreneurs, which is why the terms entrepreneur and self-employed person are used interchangeably in this study.

A comparative study by Martin (2013) between the self-employed and employees found that only around 5% of the employees surveyed work more than 10 h a day on more than 10 days a month, while this proportion is 27% for entrepreneurs (Martin, 2013). A high workload is mainly caused by customers postponing deadlines, delayed planning or buffer times when order volumes are low (Clasen, 2012, p. 99; Kottwitz et al., 2019, p. 132). In addition, the requirements associated with the high workload are diverse, highly demanding and complex (Kottwitz et al., 2019, p. 132). Entrepreneurs are also exposed to high competitive pressure (Oren, 2012, p. 168; Kottwitz et al., 2019, p. 140). In addition, entrepreneurs experience considerable pressure to take responsibility, as they often bear sole responsibility for far-reaching decisions with high risk and limited resources (White and Gupta, 2020, p. 81). Another stressor in entrepreneurial self-employment is role strain (Kottwitz et al., 2019, p. 143; White and Gupta, 2020; Vandor and Meyer, 2021). It is not only the demands of the various professional roles that cause stress – entrepreneurs are simultaneously salespeople, networkers, managers, controllers, etc. – but also the role conflict between work and family or partnership, especially for female entrepreneurs (Schonfeld and Mazzola, 2015, p. 506; Kottwitz et al., 2019, p. 143). In addition, social isolation is particularly stressful for solo self-employed workers (Schonfeld and Mazzola, 2015, p. 506; Kottwitz et al., 2019, p. 134). Furthermore, stressors in connection with customers, suppliers or other interest groups should not be underestimated (Kottwitz et al., 2019, p. 134).

When stress and strain become chronic, long-term health consequences can arise (Faltermaier, 2016, p. 117; Semmer and Zapf, 2018, pp. 25–26). Long-term effects of stress are particularly related to the functionality of stress management strategies and the ability to relax. If attempts to cope fail too often and/or the necessary relaxation does not occur after stressful events, this can lead to excessive demands and long-term stress-related damage (Semmer and Zapf, 2018, p. 26). Health-related stress consequences can be divided into physiological-somatic, psychological (cognitive-emotional) and behavioral consequences (Busch et al., 2009, pp. 22–23). The physiological or somatic consequences of stress include psychosomatic complaints such as muscle tension leading to back pain or headaches, cardiovascular problems, damage to the sensory organs (tinnitus, hearing loss), digestive problems or an increased sensation of pain (Kaluza, 2015, pp. 31–33; Faltermaier, 2016, p. 152). On a psychological level, chronic stress can lead to a reduction in cognitive performance, psychological impairments such as loss of self-esteem and mental illnesses such as depression, anxiety and burnout (Kaluza, 2015, pp. 31–33; Schaper, 2019, p. 588). Burnout is a state of complete mental, physical and emotional exhaustion that is accompanied by alienation and disenchantment with work (Burisch, 2014, p. 19; Schaper, 2019, p. 589).

In addition to the stress factors, however, self-employment also harbors some resources. The self-employed often exhibit a high level of job satisfaction, as they experience a higher degree of autonomy, competence, control, and self-realization (Clasen, 2012, p. 100; Stephan, 2018, pp. 290–293; Kottwitz et al., 2019, p. 120). The use of one’s own skills in entrepreneurial self-employment is often associated with a high sense of professional self-efficacy, which can act as an important pro-tective factor against stress (Kaluza, 2015, pp. 55–56). In addition, the experience of coherence and meaning in daily work can serve as an important protective factor against stress and health impairments (Kaluza, 2015, p. 56). Furthermore, entrepreneurs perceive their responsibility and complex tasks as intrinsically motivating to a certain extent (Martin, 2013, p. 10), while self-employment offers numerous learning and development opportunities (Clasen, 2012, p. 100).

The following section shows the extent to which these resources have a positive impact on the self-employed when dealing with stressors.

### 2.3 Stress management for the self-employed

The way in which the self-employed deal with the high demands of everyday working life (so-called coping) is decisive for whether health problems occur (Kieschke and Schaarschmidt, 2003, p. 109; Stephan, 2018, p. 301; White and Gupta, 2020, p. 87). A study by Oren (2012) compared the coping strategies of the self-employed and employees in the United States of America (Oren, 2012, pp. 164–165). The study concluded that the self-employed often use active coping strategies such as direct confrontation with the problem, while employees more often choose an avoidance strategy (Oren, 2012, p. 168). In a qualitative study by Schonfeld and Mazzola (2015) on self-employed people in the cultural and creative industries, the authors came to similar conclusions. The self-employed people surveyed used active-problem-oriented coping strategies (67:23) significantly more often than emotion-oriented coping strategies when dealing with stress (Schonfeld and Mazzola, 2015, p. 508). Schneider (2011) was able to identify the following problem-oriented coping strategies among entrepreneurs in a qualitative study: Cause and error analysis, active coping by immediately dealing with possible solutions, prioritization and structuring, planning and obtaining external support (Schneider, 2011, pp. 73–77).

An emotion-focused stress management strategy that is mentioned in research in connection with entrepreneurship is so-called “self-management.” By self-management, Neck et al. (2013) mean a process of influencing one’s thoughts, beliefs and patterns in a self-motivating way that is necessary for good performance (Neck et al., 2013, pp. 469–473). Self-leadership or “positive self-instruction” by the entrepreneur is a strategy to better manage workloads and improve organizational performance (Oren, 2012; Neck et al., 2013, p. 475; Schenk, 2017, pp. 59–62). White and Gupta also emphasize that among the company-specific coping strategies there are also those that reduce rather than increase well-being (White and Gupta, 2020, p. 83). Kieschke and Schaarschmidt (2003), who were able to identify various risk groups among entrepreneurs, also come to this conclusion. A large proportion of the entrepreneurs surveyed in their study exhibited risk pattern A, which is associated with excessive commitment as a coping strategy (Kieschke and Schaarschmidt, 2003, pp. 109–110). Work intensification or “self-exploitation” is also mentioned by Clasen and Kottwitz as a relevant characteristic of entrepreneurs (Clasen, 2012, p. 100; Kottwitz et al., 2019, p. 163). Work intensification as a dysfunctional coping strategy is explained by Krause et al. (2014) with the term “self-endangerment.” The authors define self-endangerment as “actions that serve the active and problem-oriented coping of work-related stressors, but have negative effects on health [...]” (Krause et al., 2014, p. 49).

The concept of coping is the main subject of the empirical studies in this thesis. The work is primarily based on the findings presented above on the influence of coping on (mental) health. Coping strategies that are more likely to be assigned to the dysfunctional spectrum and coping strategies that are more likely to be assigned to the functional spectrum are examined for their health-effective influence on the stress level of the self-employed.

### 2.4 Hypotheses and theoretical model

Related studies show that the self-employed primarily perceive the high quantitative workload and time pressure as well as high cognitive demands such as great (decision-making) responsibility, high flexibility requirements and diverse and complex tasks as stressors (Kottwitz et al., 2019, p. 132; Lek et al., 2020, p. 502; White and Gupta, 2020, p. 81). Based on the job demand resource model and the transactional stress theory (Lazarus, 1995, pp. 212–215; Demerouti and Nachreiner, 2019, p. 121), it is assumed that high job demands, which are perceived as stressors, affect the health of self-employed workers. In this study, mental health is operationalized by the degree of mental strain. Existing research suggests that self-employed people perceive high workload, time pressure and cognitive demands such as high decision-making responsibility, flexibility requirements and multiple tasks as stressors. Therefore, this study considers quantitative and cognitive work demands as predictors of mental health (H1_1_), controlling for perceived autonomy in the work environment, which may serve as a protective factor for the mental health of the self-employed. Perceived autonomy in everyday working life, which can be a protective factor for the mental health of the self-employed, is controlled for.

The literature review also shows that the health-related consequences of stress are influenced by the coping strategies used (e.g., Lazarus, 1966, p. 216; Skinner et al., 2003, p. 231; Carver and Connor-Smith, 2010; Perrez et al., 2011, p. 284). Coping strategies can be either health-promoting or health-damaging (Skinner et al., 2003, p. 231; Mülder et al., 2021). For this study, the concept of self-endangerment is assumed to be a health-damaging coping strategy (Krause et al., 2014). The aspects of work prolongation and presenteeism were examined as self-endangering ways of working with regard to their effect on the relationship between work demands and health. For these, the assumption of a mediation effect postulated by Krause et al. (2014) was tested (H1_2_, H1_3_).

It is assumed that health burdens are more likely to be intensified by excessive work or working despite illness and that a negative influence on mental health can be measured by exceeding physical and cognitive limits similar to type A behavior (Lazarus and Folkman, 1984, pp. 215–216; Krause et al., 2014, pp. 51–54). The health-promoting coping strategies examined in this study are positive reinterpretation and proactive coping, which are also frequently used by the self-employed (Oren, 2012; Neck et al., 2013, p. 475; Schenk, 2017). They are functional coping strategies that actively and constructively deal with the stressor and/or their own stress response (Skinner et al., 2003, p. 231; Kaluza, 2015, p. 66). Moderation effects should be tested for positive reinterpretation and proactive coping (H_4_, H1_5_).

Therefore, the following five hypotheses were derived and tested inferentially:

*H1_1_*: Work demands significantly influence mental exhaustion. The higher the perceived work demands, the higher the subjectively perceived mental exhaustion.

*H1_2_*: The amount of work significantly influences the relationship between work demands and mental exhaustion. The relationship between work demands and mental exhaustion is explained or mediated by the amount of work.

H1_3_: Presenteeism has a significant influence on the relationship between work demands and mental exhaustion. The relationship between work demands and mental exhaustion is explained or mediated by presenteeism.

*H1_4_*: Positive reframing of the stressful situation significantly influences the relationship between work demands and mental exhaustion. Positive reframing moderates the relationship between work demands and mental exhaustion to the extent that a high level of positive reframing reduces the relationship.

*H1_5_*: Proactive coping significantly influences the relationship between work demands and mental exhaustion. Proactive coping moderates the relationship between work demands and mental exhaustion to the extent that a high level of proactive coping reduces the relationship.

**Figure 1:**
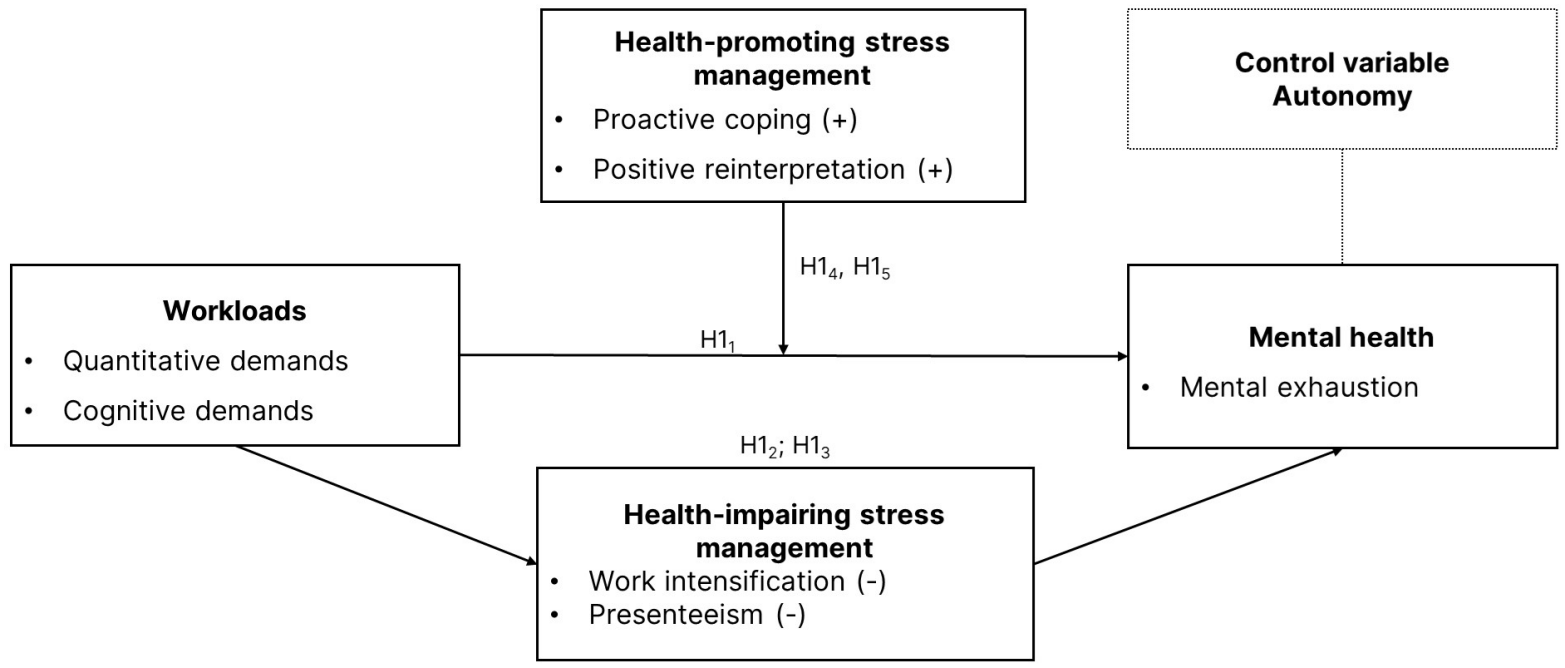
Theoretical framework. Own figure.

The hypotheses are also presented in the research model (Figure 1), which states that high job demands perceived as stressors affect the mental health of the self-employed, expressed here by the degree of mental exhaustion. This study examines the influence of coping strategies on the health effects of stress, focusing on self-endangerment as a maladaptive coping strategy among the self-employed. In particular, work extension and presenteeism are examined as potential mediators between work demands and health, as they have a negative impact on mental health. In addition, functional coping strategies such as positive reinterpretation and proactive coping are examined for their health-promoting effects in this group. Moderation effects of these strategies are also examined. The following chapter outlines the methodological approach of our study.

## 3 Methods

### 3.1 Research design

The research work is explanatory, i.e., it has a theory-and hypothesis-testing character (Döring and Bortz, 2016, pp. 192–193). In this pilot study, the theoretically derived research model was tested for the target group of self-employed persons. A quantitative, hypothesis-testing research design was chosen to test the hypotheses (Döring and Bortz, 2016, pp. 184–185). The data was collected using a fully standardized online questionnaire in a non-probabilistic cross-sectional convenience study among self-employed persons in Germany (Döring and Bortz, 2016, p. 294). The questionnaire was created using the web-based online survey software TIVIAN and made available to the participants via a corresponding link. The survey was open for participation from July 2023 to August 2023. Before the survey began, a qualitative pre-test was conducted with 7 people. The estimated completion time was approx. 10 min, based on the experience from the pretest, and was communicated to the participants in advance. In addition, all participants were informed in advance about confidentiality, anonymity and data protection regulations and had to agree to the data protection regulations.

The chosen method of data collection through an online survey harbors both opportunities and risks, which will be critically examined here. For example, only people with Internet access and a presence in the forums relevant for participant recruitment can be reached digitally. The latter poses the problem of self-selection bias. This means that the sample cannot be determined probabilistically by random selection or systematic sampling, but that the participants assign themselves to the sample (Döring and Bortz, 2016, p. 415). However, a major advantage of the online survey is its high efficiency (Döring and Bortz, 2016, p. 414). An online survey was chosen for this study due to its efficiency advantage. The link to the survey was primarily distributed via the online business network “LinkedIn,” with additional support from various organizations for dissemination. Statistical analysis was conducted using IBM SPSS, with the mediation and moderation hypotheses tested using (Hayes, 2013) PROCESS macro, which uses bootstrapping.

### 3.2 Variables

For the measurement of latent variables, validated instruments were selected according to criteria such as test quality, availability of German scales and realistic survey time. In the following section, these instruments and their test quality criteria are presented, with a focus on internal consistency and construct validity. Reliability measures (test–retest reliability r_tt; Cronbach’s alpha α) above 0.70 are considered acceptable, while correlations with *r* = 0.10, 0.30 and 0.50 are interpreted as small, moderate and large, respectively, (Nunnally, 1967, 1978; Cohen, 1988). The following variable schedule contains the scales used, see Table 1.

**Table 1:** Dimensions and indicators.

Dimension	Scales	Authors
*Independent variables* Workload Quantitative requirements Cognitive requirements	Scales for cognitive and quantitative requirements of the COP-SOQ in German version	Modified according to Nübling et al. (2005)
*Dependent variable* Mental health	Scale for personal burnout/exhaustion from the CBI	Modified according to Kristensen et al. (2005) in German translation in the COPSOQ (see Nübling et al., 2005)
*Mediator/moderator variables* Health-promoting stress management Proactive Coping Positive reinterpretation Health-impairing stress management Work intensification Presenteeism	Scale for positive reinterpretation and proactive coping from the Stress Coping Inventory (SCI) Scale for extending working hours and scale for presenteeism from the survey instrument for recording self-endangerment	Modified according to Satow (2012) According to Krause et al. (2014)
*Control variable* Autonomy	Autonomy scale from the measurement model for basic need satisfaction at work	According to Koch and Zunk (2021) (German translated)

Dimensions and indicators used in the study (Kristensen et al., 2005; Nübling et al., 2005; Satow, 2012; Krause et al., 2014; Koch and Zunk, 2021).

#### 3.2.1 Copenhagen psychosocial questionnaire

All scales used were adapted to the purpose of the study and tested. Perceived workload was assessed using the “Quantitative demands” and “Cognitive demands” scales of the Copenhagen Psychosocial Stress Questionnaire (COPSOQ) in its German version. The original Danish version was further developed to measure psychosocial workload. The COPSOQ is used both as a screening instrument in practice and for research and evaluation purposes in science (Nübling et al., 2005, p. 12). It consists largely of measurement instruments that have already been tested and validated (Nübling et al., 2005, p. 13).

#### 3.2.2 Copenhagen burnout inventar

In the original study with a sample size of *N* = 1898, a high internal reliability of α > 0.87 was achieved for the personal burnout scale (Kristensen et al., 2005, p. 200). Similarly, in the German translation of the COPSOQ by Nübling et al. (2005), a high reliability of *α* > 0.90 was achieved for the personal burnout scale with six items. Construct validity was tested in the original study using the SF-36 Health Survey. As expected, high correlations were found between the “Vitality” and “Mental Health” scales of the SF-36 and the personal burnout scale (*r* = 0.75; *r* = 0.67) (Kristensen et al., 2005, p. 202). In the German study on the translation of the COPSOQ, the personal burnout scale showed high correlations in the expected direction compared to similar outcome constructs such as job satisfaction (Nübling et al., 2005, p. 56). It can therefore be assumed that the scale has high construct validity.

#### 3.2.3 Stress and coping inventory

The scales for the latent variables of proactive coping and positive reframing as health-promoting coping strategies were taken from the Stress and Coping Inventory (SCI) by Satow (2012). The SCI assesses five coping strategies, including positive thinking and proactive coping, using a self-assessment questionnaire (Satow, 2012, p. 6). The SCI was selected for this study because it offers a comprehensive scale, high-quality documentation and free availability.

#### 3.2.4 Instrument for measuring self-harm

The variables for the coping strategies of extended working hours and presenteeism were taken from the instrument for recording self-harm by Krause et al. (2014). The scales on working time extension and presenteeism are two of eight facets of self-harm that were identified in a qualitative preliminary study (Krause et al., 2014, p. 52). Extending one’s own working hours primarily refers to the time limit on work, while presenteeism is understood to mean working despite illness or foregoing regeneration during illness (Krause et al., 2014, pp. 52–53).

#### 3.2.5 Scale for basic needs satisfaction in the workplace

The control variable autonomy was measured using the autonomy scale from the measurement instrument “Scale for basic needs satisfaction in the workplace” (Koch and Zunk, 2021, pp. 13–14). Construction and response format. The German-language scale on autonomy comprises 6 items, which have a 5-point Likert scale in the German-language translation (Koch and Zunk, 2021, pp. 20–21).

### 3.3 Sample

The sample consisted of 117 participants drawn from the population of self-employed people in Germany (Döring and Bortz, 2016). Following the ICSE, we define an entrepreneur or self-employed person as a natural or legal person who acts independently in the exercise of their commercial or professional activity, which includes both self-employed persons and persons with employees, as defined in the German Civil Code (see section 2.2). According to the Federal Statistical Office, there were around 3.887 million self-employed persons in Germany in the first quarter of 2023 (Federal Statistical Office, 2023a; Federal Statistical Office, 2023b). Of the 839 people who accessed the survey link, 130 completed the questionnaire, which corresponds to a completion rate of 15.49%.

After data cleansing, the data set comprised 117 participants (*n* = 117). Two filter variables were used at the beginning of the questionnaire to refine the sample. Thirteen participants who stated that they were not employed or not self-employed were excluded from the sample. Of the 117 participants, 58 (49.6%) reported being male, 59 (50.4%) reported being female, and no participants described themselves as non-binary. Fifty-nine participants (50.4%) reported having employees, while 58 (49.6%) were solo self-employed. The average age of participants was 42.26 years (*M* = 42.26, SD = 11.10), with the youngest participant being 21 years old and the oldest 66 years old. Half of the participants were under 40 years old, and 25% were younger than 33.5 years. On average, participants had 11.95 years of self-employment experience (*M* = 11.95, SD = 10.61), with three individuals having 40 years of self-employment experience. Further demographic information can be found in Table 2.

**Table 2:** Demographic data (*n* = 117).

Category	Frequency	Percentage
*Sex*		
Female	59	50,4
Male	58	49,6
*Form of entrepreneurship*		
Employer (employs workers)	59	50.4
Solo self-employed	58	49.6
*Level of experience*		
0–5	46	39,3
6–11	20	17,1
12–17	20	17,1
≥18	31	26,5
*Age*		
18–28	11	9,4
29–38	39	33,3
≥ 39	67	57,3

### 3.4 Data analysis

The statistical analysis was carried out using the statistical program IBM SPSS. The SPSS macro procedure by Hayes (2013), which is based on bootstrapping, was used to test the mediator and moderator hypotheses. The statistical methods of linear regression, moderation and mediation, which are briefly explained below, were used to empirically test the hypotheses described.

#### 3.4.1 Linear regression

Hypothesis H1_1_ postulates a negatively directed, semi-partial association hypothesis (Döring and Bortz, 2016, pp. 146, 666–667, 679). Association hypotheses can be divided into bivariate, partial and multiple association hypotheses (Döring and Bortz, 2016, p. 678). A bivariate association hypothesis examines the relationship between two variables: an independent predictor variable and a dependent criterion variable (Döring and Bortz, 2016, pp. 146, 678). In the case of a partial association hypothesis, the influence of a control variable on the criterion variable is also eliminated (semi-partial correlation) (Döring and Bortz, 2016, p. 678). A multiple association hypothesis also examines relationships between a criterion and several predictors (Döring and Bortz, 2016, pp. 146, 678). Association hypotheses such as H1_1_ are tested using linear regression analysis. Linear regression aims to predict the development of an outcome variable (criterion) under the influence of one or more independent variables (predictors) (Leonhart, 2013, pp. 311–312, 338).

#### 3.4.2 Moderation

In (multiple) linear regression, the focus of the analysis is on the individual predictors of a regression model and their effects on the criterion. It is also possible to analyze interaction effects of variables on an existing relationship. The interaction effect is also referred to as moderation (Field, 2013, p. 395; Hayes, 2013, pp. 208–209; Bühner and Ziegler, 2017, pp. 769–770). Hypotheses H1_4_ and H1_5_ represent moderation hypotheses.

#### 3.4.3 Mediation

Hypotheses H1_2_ and H1_3_ represent mediation hypotheses. Mediation occurs when the relationship between predictor and criterion is influenced by an intervening third variable (mediator M) (Hayes, 2013, pp. 86–87; Bühner and Ziegler, 2017, p. 760). In contrast to the moderator variable, which influences the extent of the relationship between X and Y, the mediator variable establishes the relationship between X and Y (Bühner and Ziegler, 2017, p. 760).

## 4 Results

### 4.1 Descriptive values

In research practice, Cronbach’s α is usually used as a coefficient to determine reliability. Values of at least 0.70 are considered sufficient, values of 0.80 are recommended and values above 0.90 are considered high [Nunnally, 1967, 1978, cited in Gäde et al. (2020), p. 331; Janssen and Laatz, 2017, p. 611]. As validated and reliable measurement instruments were used to operationalize the variables, albeit with adapted response formats. Reliability analyzes revealed acceptable to very good reliability values for all scales despite the modified response format (Cronbach’s α between 0.72 and 0.92), which indicates good study quality. For none of the scales was there a clear improvement in Cronbach’s α due to the exclusion of one or more items, so that no items were excluded from the analysis.

Table 3 provides an overview of the descriptive values of our analysis. For the scales describing job requirements, the mean value for quantitative requirements was 3.77 (standard deviation SD = 0.98) and for cognitive requirements 4.74 (SD = 0.644). The level of cognitive requirements was therefore higher on average than that of quantitative requirements. The overall index of requirements had a mean value of 4.29 (SD = 0.64). In the case of quantitative requirements, 44.4% of respondents stated that they were often to always confronted with these requirements, while the percentage for cognitive requirements was 88.0%. On the scales for health-promoting stress management, the scale for positive reinterpretation/reframing had a mean value of 3.86 (SD = 0.92) and the scale for proactive coping had a mean value of 3.97 (SD = 1.00). On a scale of 1–6, these values correspond to moderately pronounced tendencies. In the scales on health-damaging stress management, the scale on work prolongation had a mean value of 3.69 (SD = 0.98), and the scale on presenteeism had a mean value of 2.45 (SD = 1.35). 28.2% of respondents stated that they rarely or never extend their working hours, while 71.8% stated that they rarely or never work when they are ill. The extent of extended working hours was therefore moderate, while the extent of presenteeism was relatively low. The scale for mental exhaustion resulted in a mean value of *M* = 3.22 (SD = 0.96) in the medium range. 16.2% of respondents stated that they were often to always mentally exhausted. The control variable autonomy showed a relatively high level with a mean value of 4.56 (SD = 0.70).

**Table 3:** Scale means, standard deviations, and Cronbach’s α of the variables (*n* = 117).

Theoretical constructs	Variables	*M*	*SD*	*α*
	Age	42.26	11.10	/
	Level of professional experience	11.95	10.61	/
Workloads	Quantitative demands	3.77	0.98	0.88
	Cognitive demands	4.74	0.64	0.72
	Demands total (index)	4.29	0.64	0.83
Health-promoting stress coping	Positive reframing/reframing	3.86	0.92	0.78
	Proactive Coping	3.97	1.00	0.87
Health-impairing stress coping	Work extension	3.69	0.98	0.78
	Presenteeism	2.45	1.35	0.92
Mental health	Mental exhaustion	3.22	0.96	0.89
Control variable	Autonomy	4.56	0.70	0.73

A Likert scale of 1 to 6 was used (1 = does not apply at all; 6 = applies completely). Values in the table have been rounded to two decimal places.

The data show that only 16.2% of participants stated that they were often to always mentally exhausted. This contrasts with the findings of Clasen (2012), according to which one in five freelancers suffered from severe mental stress. However, the cognitive demands were particularly high: 88.0% were often to always confronted with them. Autonomy, which is inversely correlated with both quantitative demands (*r* = −0.44, *p* < 0.001) and mental exhaustion (*r* = −0.45, *p* < 0.001), proved to be a significant protective factor for the mental well-being of freelancers (Clasen, 2012, p. 100; Oren, 2012, p. 164). Proactive coping was rated moderately high (*M* = 3.97, SD = 1.00), while presenteeism was low (*M* = 2.45, SD = 1.35), possibly due to an increased awareness of the negative effects during the Covid-19 pandemic. Nevertheless, the responses could be influenced by social desirability after the pandemic. A weak but significant positive correlation was found between presenteeism and work experience (*r* = 0.20, *p* = 0.030), which raises the question of why long-standing self-employed workers might overlook health concerns compared to new entrants. This tendency could be due to the increasing responsibility and greater consequences of absenteeism with longer periods of self-employment.

### 4.2 Hypothesis tests overview

All hypotheses were tested with a significance level of at least α < 0.05. The analyses are all based on a 95% confidence interval (CI). A summary of the hypothesis tests can be found in Table 4.

**Table 4:** Results of the hypothesis tests.

Hypothesis		Results
H0_1_	Work demands (overall index) have no significant influence on mental exhaustion.	Rejected
H1_1_	Work demands have a significant influence on mental exhaustion. The higher the perceived work demands, the higher the subjectively perceived mental exhaustion.	Accepted
H0_2_	The amount of work has no significant influence on the relationship between work demands and mental exhaustion.	Rejected
H1_2_	The amount of work has a significant influence on the relationship between work demands and mental exhaustion. The relationship between work demands and mental exhaustion is explained or mediated by workload.	Accepted
H0_3_	Presenteeism has no significant influence on the relationship between work demands and mental exhaustion.	Rejected
H1_3_	Presenteeism has a significant influence on the relationship between work demands and mental exhaustion. The relationship between work demands and mental exhaustion is explained or mediated by presenteeism.	Accepted
H0_4_	Positive reinterpretation has a significant influence on the relationship between work demands and mental exhaustion.	Accepted
H1_4_	Positive reinterpretation significantly influences the relationship between work demands and mental exhaustion. Positive reinterpretation moderates the relationship between work demands and mental exhaustion, in that a high degree of positive reinterpretation reduces the relationship.	Not confirmed
H0_5_	Proactive coping has no significant influence on the relationship between work demands and mental exhaustion.	Accepted
H1_5_	Proactive coping significantly influences the relationship between work demands and mental exhaustion. Proactive coping moderates the relationship between work demands and mental exhaustion, in that a high level of proactive coping reduces the relationship.	Not confirmed

Own representation.

#### 4.2.1 Hypothesis H1_1_

In H1_1_, multiple linear regression was used to test the extent to which the individual requirement components quantitative requirements and cognitive requirements influence mental exhaustion. The influence of the control variable autonomy on mental exhaustion was controlled. A hierarchical regression was calculated. In the first stage, the control variable autonomy was included in the model (model 1); in the second stage, the predictors quantitative demands and cognitive demands were also included (model 2). Table 5 shows all relevant coefficients and measured values for hypothesis testing. A highly significant influence on mental exhaustion was found for the predictor variable quantitative demands after inclusion of all variables (model 2) (*B* = 0.38, β = 0.39, *p* < 0.001). For the predictor variable cognitive demands, there was no significant influence on mental exhaustion after including all variables (model 2) (*B* = −0.03, *β* = −0.02, *p* = 0.782). Model 2 was highly significant and explained 33% of the variance in mental exhaustion (*R*^2^ = 0.33, corrected *R*^2^ = 0.31, *F*(3, 113) = 18.16, *p* < 0.001). After including the predictors cognitive and quantitative demands in model 2, a further 12% of the variance in mental exhaustion could be significantly explained by the control variable autonomy compared to model 1 (Δ*R*^2^ = 0.12, Δ*F* = 10, *p* < 0.001). If the autonomy variable is controlled, the cognitive and quantitative demands can still explain 12% of the variance in the mental exhaustion criterion.

**Table 5:** Regression models with predictors quantitative and cognitive demands, control variable autonomy, and criterion mental exhaustion (*n* = 117).

	Mental exhaustion					
	Model 1			Model 2		
Variable	*B*	*β*	*t*	*B*	*β*	*t*
Autonomy	−0.62^**^	−0.45^**^	−5.47	−0.39^**^	−0.29^**^	−3.90
Demands quantitative	–			0.38^**^	0.39^**^	4.40
Demands cognitive				−0.03	−0.02	−0.28
*R* ^2^	0.21^**^0.33^**^					
*F*	29.90^**^ 18.16^**^					
Δ*R*^2^	0.12^**^					
Δ*F*	0.10^**^					

Β, Regression coefficient; β, standardized regression coefficient. *p < 0.05, ** *p* < 0.01.

#### 4.2.2 Hypothesis H1_2_

Hypothesis H1_2_ assumes a mediating relationship in which the expansion of work explains the relationship between general work demands and mental exhaustion. In testing this hypothesis, the influence of autonomy was also controlled for, see Table 6. The mediator analysis, conducted using the SPSS macro PROCESS version 4.2 by Andrew Hayes, revealed a significant overall effect of job demands on mental exhaustion (*B* = 0.44, *β* = 0.30, *p* = 0.01). The overall effect of job demands significantly predicted work prolongation (*B =* 0.79, β = 0.52, *p* < 0.001), which in turn significantly predicted mental exhaustion (*B =* 0.30, β = 0.30 *p* = 0.001). The indirect effect (axb) of general job demands on mental exhaustion due to job enlargement was significant (*B* = 0.23, 95% CI = [0.0866, 0.3944], β = 0.16, 95% CI = [0.0570, 0.2616]). After including the mediator in the model, the direct effect of total work demands on psychological exhaustion was no longer significant (*B =* 0.21, β = 0.14, *p* = 0.144). Thus, H0_2_ is rejected, while H1_2_ is accepted.

**Table 6:** Regression coefficients B, standard errors, and *t*-values for the mediator model for H1_2_.

		Results of the paths and effect sizes								
		*M* work extension						*Y* mental exhaustion		
Variable		*B*	β	*SE*	*t*		*B*	β	*SE*	*t*
*X* (ANF_Index)	*a*	**0.79** ^**^	**0.52** ^**^	0.13	6.12	*c`*	0.21	0.14	0.14	1.47
*M* (ABAD)		–	–	–	–	*b*	**0.30** ^**^	**0.30** ^**^	0.09	3.30
Constant	*i_1_*	0.91	–	0.91	1.0	*i_2_*	**3.15** ^**^	–	0.88	3.59
		*R*^2^ = 0.31^**^ *F*(2, 114) = 26.04^**^, *p* < 0.001					*R*^2^ = 0.34^**^ *F*(3, 113) = 19.77^**^, *p* < 0.001			

*p < 0.05; **p ≤ 0.01. ANF_Index, Total job demands index; EXT, Work extension; EX, Mental exhaustion. Coefficients (B, β) at a significance level of at least *p* < 0.05 are highlighted in bold.

#### 4.2.3 Hypothesis H1_3_

Hypothesis H1_3_ suggests that presenteeism mediates the relationship between general job demands and mental exhaustion (Hayes, 2013, pp. 86–87; Bühner and Ziegler, 2017, p. 760). When testing the hypothesis, the influence of autonomy was controlled. The mediating effect was calculated with SPSS Macro PROCESS by Hayes, whereby the entire model took the autonomy variable into account. A significant overall effect was found between general job demands and psychological exhaustion (*B =* 0.44, β = 0.30, *p* < 0.001). A significant direct effect of general job demands on presenteeism (*B =* 0.71, β = 0.34, *p* = 0.001) and of presenteeism on mental exhaustion (*B =* 0.23, β = 0.32, *p* = 0.001) was observed. The indirect effect of general job demands on mental exhaustion (controlled for autonomy) by presenteeism was significant (*B =* 0.16, 95% CI = [0.0478, 0.3069], β = 0.11, 95% CI = [0.0316, 0.2024]). Even after including the mediator, the direct effect of general job demands on mental exhaustion (controlled for autonomy) remained significant at a 5% level (*B =* 0.28, β = 0.19, *p* = 0.031), indicating partial mediation.

H1_3_ is accepted, and H0_3_ is rejected. The regression coefficients, standard errors, and t-values for the model are shown in Table 7.

**Table 7:** Regression coefficients B, standard errors, and *t*-values for the mediator model for H1_3_, see Figures 19 and 20 (*n* = 117).

		Results of the paths and effect sizes								
		*M* presenteeism					*Y* mental exhaustion			
Variable		*B*	β	*SE*	*t*		*B*	β	*SE*	*t*
*X* (ANF_Index)	*a*	**0.71** ^**^	**0.34** ^**^	0.19	3.75	*c`*	**0.28** ^*^	**0.19** ^*^	0.13	2.19
*M* (PRAES)		–	–	–	–	*b*	**0.23** ^**^	**0.32** ^**^	0.06	3.75
Constant	*i_1_*	1.35	–	1.34	1.0	*i_2_*	**3.11** ^**^	–	0.87	3.59
		*R*^2^ = 0.22^**^ *F*(2, 114) = 16.17^**^, *p* < 0.001					*R*^2^ = 0.36^**^ *F*(3, 113) = 21.24^**^, *p* < 0.001			

*p < 0.05; **p ≤ 0.01.

ANF_Index, Total job demands index; PRESE, Presenteeism; EX, Mental exhaustion Coefficients (B, β) at a significance level of at least *p* < 0.05 are highlighted in bold.

#### 4.2.4 Hypothesis H1_4_

Hypothesis H1_4_ examines interaction effects and functions as a moderator hypothesis. It tests the effects of positive reframing on the relationship between general job demands and mental exhaustion, controlling for autonomy variables. Table 8 contains the results of the regression analysis, which examines the moderation effect of positive reframing on the relationship between general job demands and mental exhaustion. After including the product term Z in the regression model, no significant interaction effect of positive reframing (centered) on the relationship between general job demands (centered) and mental exhaustion, controlling for the autonomy variable (centered), was found (*B* = −0.21, β = −0.12, *p* = 0.131). H0_4_ is accepted, and H1_4_ is not confirmed.

**Table 8:** Results of the regression analysis examining the moderation effect of positive reframing on the relationship between overall job demands index and mental exhaustion (*n* = 117).

Variables		Mental exhaustion							
		Model 1				Model 2			
		*B*	β	*SE*	*t*	*B*	β	*SE*	*t*
Constant		**3.22** ^**^	−	0.74	43.28	**3.23** ^**^	−	0.07	43.48
ANF Index (X´)	*b*_1_	**0.47** ^**^	**0.32** ^**^	0.13	3.69	**0.48** ^**^	**0.32** ^**^	0.13	3.76
REF (M´)	*b*_2_	**−0.18** ^*^	**−0.17** ^*^	0.08	−2.14	**−0.18** ^*^	**−0.18** ^*^	0.08	−2.26
Product term (X´M´)	*b*_3_	−	−	−	−	−0.21	−0.12	0.14	−1.52
AUT (C´)	*b_4_*	**−0.45** ^**^	**−0.33** ^**^	0.12	−3.86	**−0.44** ^**^	**−0.32** ^*^	0.12	−3.83
*R^2^*		**.31** ^**^				**.32** ^**^			
*F*		**16.85** ^**^				**13.37** ^**^			
Δ*R^2^*						.01			
Δ*F*						2.31			

*p < 0.05, **p ≤ 0.01.

ANF_Index (X´), The overall job demands index centered; REF (M´), centered positive reframing; Product term Z, centered overall job demands index × centered positive reframing (X’M´). The variables overall job demands index (X´), positive reframing (M´), overall job demands index × positive reframing (X’M´), and the control variable AUT (C´) are centered around the mean. Coefficients (B, β) at a significance level of at least *p* < 0.05 are highlighted in bold.

In addition, significant positive effects of the predictor overall job demands (centered) and negative effects of the moderator reframing (centered) were observed in both models. Overall job demands (centered) had a significant positive effect on mental exhaustion (*B* = 0.47, β = 0.32, *p* < 0.001 or *B* = 0.48, β = 0.32, *p* < 0.001). The reframing moderator (centered) had a significant negative effect on mental exhaustion at the 5% level in both models (*B* = −0.18, β = 0.17, *p* = 0.034 or *B* = −0.18, β = −0.18, *p* = 0.026 Model 1, which included general job demands, reframing, and autonomy (each centered), significantly explained 31% of the variance in mental exhaustion (*R^2^* = 0.31, adjusted *R*^2^ = 0.27, *F*(3, 113) = 16.85, *p* < 0.001) = 16.85, *p* < 0.001). The inclusion of the product term Z in the model did not lead to a significant change in the explanation of variance (Δ*R*^2^ = 0.01, Δ*F* = 2.31, *p* = 0.131).

#### 4.2.5 Hypothesis H1_5_

Hypothesis H1_5_, also a moderator hypothesis, tests the effect of proactive coping (ProCop) on the relationship between general work demands and mental exhaustion, controlling for the autonomy variables. The results in Table 9 show the results of the regression analysis examining the modulation effect of proactive coping on the relationship between general work demands and mental exhaustion. After including the product term Z in the regression model, no significant interaction effect of proactive coping (centered) on the relationship between general work demands (centered) and mental exhaustion, controlling for the autonomy variable (centered), was found (*B* = −0.01, *β* = −0.00, *p* = 0.968). H0_5_ is accepted, and H1_5_ is not confirmed.

**Table 9:** Results of the regression analysis examining the moderation effect of proactive coping on the relationship between overall job demands index and mental exhaustion (*n* = 117).

		Mental exhaustion							
		Model 1				Model 2			
Variables		*B*	β	*SE*	*t*	*B*	β	*SE*	*t*
Constant		**3.22** ^**^	–	0.07	43.29	**3.22** ^**^	–	0.08	42.05
ANF Index (X´)	*b*_1_	**0.40** ^**^	**0.27** ^**^	0.13	3.14	**0.40** ^**^	**0.27** ^**^	0.13	3.12
PROCop (M´)	*b*_2_	**−0.17** ^*^	**−0.18** ^*^	0.08	−2.16	−0.17^*^	**−0.18** ^*^	0.08	−2.08
Product term (X’M´)	*b*_3_	–	–	–	–	−0.01	−0.00	0.12	−0.04
AUT (C´)	*b_4_*	**−0.42** ^**^	**−0.31** ^**^	0.12	−3.59	**−0.42** ^**^	**−0.31** ^**^	0.12	−3.55
*R^2^*		**0.31** ^**^				**0.31** ^**^			
*F*		**16.89** ^**^				**12.56** ^**^			
Δ*R^2^*						0.00			
Δ*F*						0.00			

*p < 0.05, **p ≤ 0.01.

ANF_Index (X´), Overall job demands index centered; POS (M´), centered positive reframing; product term Z, JOB Index × POS (X’M´); the variables JOB Index (X´); POS (M´), JOB Index × POS (X’M´), and the control variable AUT (C´) are centered around the mean. Coefficients (B, β) at a significance level of at least *p* < 0.05 are highlighted in bold.

In addition, significant positive effects of the predictor general work demands (centered) and negative effects of the moderator proactive coping (centered) were observed in both models. The general work demands (centered) had a significant positive effect on mental exhaustion (*B* = 0.40, β = 0.27, *p* = 0.002 and *B* = 0.40, β = 0.27, *p* = 0.002). The proactive coping moderator (centered) had a significant negative effect on mental exhaustion at the 5% level in both models (*B* = −0.17, β = 0.18, *p* = 0.033 or *B* = −0.17, β = 0.18, *p* = 0.040). Model 1, which includes general work demands, proactive coping and autonomy (each centered), significantly explained 31% of the variance in mental exhaustion (*R*^2^ = 0.31, adjusted *R*^2^ = 0.29, *F*(3, 113) = 16.89, *p* < 0.001). The inclusion of the product term Z in the model did not lead to a significant change in the variance explanation (Δ*R*^2^ = 0.00, Δ*F* = 0.00, *p* = 0.968).

## 5 Discussion

The first research question, which is derived from the JD-R model, investigated the effects of specific work demands on the mental health of self-employed people, measured in terms of subjectively perceived mental exhaustion. After rejecting the null hypothesis, which postulated no relationship between work demands and mental exhaustion, the alternative hypothesis was confirmed, which showed a statistically significant influence of work demands on mental exhaustion (*B* = 0.44, β = 0.30, *p* = 0.001). The analysis showed that quantitative demands had a highly significant impact on mental exhaustion (*B* = 0.38, β = 0.39, *p* < 0.001), with the inclusion of this variable explaining an additional 12% of the variance in mental exhaustion. These results are consistent with recent studies that identify long working hours and time pressure as detrimental conditions for the mental health of the self-employed. Conversely, this study found no effects of cognitive demands such as high responsibility or flexibility requirements on mental exhaustion. Cognitive demands can activate other protective factors that positively influence mental health (Clasen, 2012; Martin, 2013; Kaluza, 2015).

The second research question investigated how self-endangering coping strategies such as work prolongation and presenteeism influence the relationship between work demands and mental health. Both mediator hypotheses (H1_2_ and H1_3_) were confirmed, with work extension fully mediating the relationship and presenteeism partially mediating it. The results indicate that high job demands can lead to self-endangering behaviors that impair mental health. These results confirm similar studies in Switzerland and underline the importance of self-harm coping strategies for the self-employed and entrepreneurs (see Dorsemagen et al., 2012; Krause et al., 2012, pp. 198–199; Krause et al., 2014, p. 51).

The third research question investigated the health-promoting effect of coping strategies such as positive reframing and proactive coping. The hypotheses regarding moderating effects (H1_4_ and H1_5_) could not be confirmed. Neither positive reframing nor proactive coping showed a significant moderating effect on the relationship between work demands and mental exhaustion. The discrepancy in the definition of the reframing coping strategy and in the measurement instruments may affect the comparability of the study results. However, significant negative correlations were found between proactive coping and work demands and mental exhaustion. These results confirm previous studies and suggest that proactive coping primarily reduces work demands and thus contributes to lower mental exhaustion.

The results are in line with the assumptions of the JD-R model by Bakker and Demerouti, which have already been empirically confirmed in several studies (Bakker et al., 2004; Bakker and Demerouti, 2007, pp. 315–317). When analyzing the individual components of work demands (cognitive and quantitative demands), a highly significant influence of quantitative demands on mental exhaustion was found (*B* = 0.38, β = 0.39, *p* < 0.001). After including the variable quantitative demands, a highly significant 12% more variance in mental exhaustion could be explained. This is in line with recent studies that have identified a high workload and the associated long working hours and time pressure as harmful conditions for the mental and physical health of the self-employed (e.g., Stephan, 2018; Kottwitz et al., 2019, p. 120; Lek et al., 2020, pp. 502–503).

In contrast to current research, however, no influence of cognitive demands such as high responsibility or flexibility requirements on mental exhaustion was found in this study (*B* = −0.03, *β* = −0.02, *p* = 0.78; e.g., Kottwitz et al., 2019, pp. 132–133; Lek et al., 2020, p. 502; White and Gupta, 2020, pp. 80–81). One possible explanation for this could be that cognitive demands such as high responsibility, pressure to innovate or flexibility in turn activate other protective factors such as high self-efficacy, intrinsic motivation or meaningfulness of work, which have a positive effect on mental health (Clasen, 2012, pp. 99–10; Martin, 2013, p. 10; Kaluza, 2015, pp. 55–56). In addition, with regard to the person-environment fit model (P-E fit model), it can be hypothesized that the self-employed respondents have relevant personality traits, goals and values that correspond to the cognitive requirements of self-employment (Oren, 2012, p. 168).

The role of the control variable autonomy in the model was also interesting. Autonomy was highly significant in explaining 21% of the variance in mental exhaustion and had a negative effect on mental exhaustion (*B* = −0.62, β = −0.45, *p* < 0.001). In line with existing research findings, it can be concluded that autonomy is an important resource for the self-employed and a protective factor for the mental health of the self-employed (e.g., Oren, 2012, p. 164; Stephan, 2018, p. 293; Kottwitz et al., 2019, p. 120).

The two mediator hypotheses (H1_2_ and H1_3_) were also confirmed: Both work duration and presenteeism showed a mediating effect between work demands and mental exhaustion among the self-employed respondents in this study. The results show that high work demands can lead to self-endangering behavior among the self-employed respondents, which in turn impairs their mental health. Self-endangering coping strategies are therefore of great importance in the context of self-employment and entrepreneurship and, as assumed in the research model of this study, can certainly be described as harmful to health. Self-endangering behaviors, as described in risk pattern A by Kieschke and Schaarschmidt (2003), are widespread among business founders according to their findings. These behaviors therefore appear to be a critical factor for the mental health of the self-employed (Kieschke and Schaarschmidt, 2003, p. 110). It is reasonable to conclude that many entrepreneurs tend toward a type A risk behavior pattern that is associated with self-endangering behavior, which in turn leads to mental exhaustion in connection with the high demands of self-employment.

Moderating effects were assumed in the hypotheses (H1_4_ and H1_5_). However, both moderator hypotheses could not be confirmed. Contrary to the hypotheses of Neck et al. (2013), no significant moderator effect of the coping strategy positive reinterpretation/reframing on the relationship between work demands and mental exhaustion was found (product term Z: *B* = −0.21, *β* = −0.12, *p* = 0.131) (Neck et al., 2013, pp. 469–474). No significant moderating effect was found for the coping strategy proactive coping on the relationship between work demands and mental exhaustion (product term Z: *B* = −0.01, *β* = −0.00, *p* = 0.968). However, significant negative correlations were found between proactive coping and demands (*r* = −0.22, *p* = 0.016) and between proactive coping and mental exhaustion (*r* = −0.31, *p* = 0.001).

The results of our pilot study thus confirm previous research: a negative correlation between proactive coping and mental exhaustion has already been demonstrated in several studies (Ângelo and Chambel, 2014, p. 205). Here, too, it can be argued at this point that proactive coping reduces the demands of advance, which in turn contributes to lower mental exhaustion. In addition, a significant correlation was also found between proactive coping and autonomy (*r* = 0.23, *p* = 0.015). This expression may support Oren’s (2012) assumption that proactive coping is more likely to be used in autonomous work environments, as it is more helpful there than in work environments where the employee has less control over the organization of their work (Oren, 2012, p. 168). However, it can also be the other way around if the employer creates scope for autonomous action through careful planning and early processing of potential stressors.

### 5.1 Research limitations

A quantitative study design with a small sample was used in this study. It is therefore necessary to critically reflect on the data collection and analysis with regard to the following aspects: Sample size and representativeness, validity criteria of the instruments used and scope of the study. One incomprehensible criticism concerns the representativeness of the sample, which was not probabilistic and self-selected (Döring and Bortz, 2016). The survey was distributed online and offline through various channels and regional networks, which allowed for voluntary participation. This method limited the sample to people with access to these networks and a basic interest in the topic, which may have led to an underrepresentation of skeptical groups (Döring and Bortz, 2016). It was therefore not possible to form a representative sample that reflects the characteristics of the population. Furthermore, due to the cross-sectional design of the study, no causal relationships can be derived, meaning that the assumed directions of the associations cannot be empirically tested. However, these assumed relationships were logically derived from theory, which is a strength of this study. In addition, some socio-demographic and occupational characteristics were evenly distributed, which mitigated biases due to gender or employment status. However, the study lacked industry-specific data, which could lead to bias. Therefore, this study is a pilot study that requires further research (see below).

Another limitation concerns the limited extent to which the construct of coping was statistically analyzed. Coping is a complex, multidimensional construct with different styles and strategies. Due to time and resource constraints, only four main coping strategies were examined. However, this study does not claim to comprehensively capture the coping behavior of the self-employed. Validated instruments in the literature provide comprehensive assessments of coping styles, but their high number of items would have been impractical for this study. By taking these considerations into account, the study remains transparent and highlights areas for improvement and further research.

### 5.2 Further research

The results of this study suggest several avenues for further research. In addition to examining specific assumptions, such as the mediating effect of proactive coping strategies, there is a need for an expanded research model. As mentioned earlier, the coping constructs were not fully explored in this study. Therefore, future studies should comprehensively investigate the coping strategies of self-employed individuals using existing measurement instruments. Qualitative research designs could also provide insights into the nuanced coping strategies of the self-employed. In addition, it is recommended to include aspects of entrepreneurial personality in the research model. Personal factors are closely related to the choice of coping strategies, and understanding how entrepreneurial characteristics influence coping strategies and consequently mental health is a valuable research approach. In addition, examining the dual pathways of the JD-R model— specifically the relationship between occupational resources, engagement and psychological exhaustion in the self-employed — could provide valuable insights into entrepreneurial success. In addition, future research should consider a broader range of stressors within a single model and examine industry classifications to identify vulnerable groups in need of special support.

In terms of practical implications, the findings suggest various strategies for entrepreneurship centers and public or private programs to promote entrepreneurship. These institutions could implement psychoeducational programs and stress management workshops to provide entrepreneurs with effective coping strategies for the demands of self-employment. Educating entrepreneurs about the health risks associated with self-endangering behavior is also critical. Offering voluntary risk assessments or aptitude diagnoses as part of entrepreneurial coaching can help individuals make informed career decisions or seek appropriate support from the outset. In addition, tailored mental health support services should be offered to prevent incapacity and ensure business continuity. In the context of solo self-employment, it could be beneficial to incentivize client companies to prioritize the health of their contractors. For example, client companies could offer their contractors the opportunity to participate in their company health programs.

## 6 Conclusion

In this study, we provide an insight into the stress factors that entrepreneurs face and the coping strategies they use to deal with them. It has been confirmed that self-endangering coping behavior (work prolongation and presenteeism) has a negative impact on mental health. In self-employed workers, proactive coping did not mitigate the link between work demands and mental exhaustion. Based on the key questions of this study, it becomes clear that high job demands in entrepreneurial or self-employed activities can indeed have critical effects on the mental health of self-employed people. Quantitative demands such as time pressure and workload are particularly relevant. In addition, the use of stress management strategies by the self-employed plays an important role in this context. Although it was not possible to collect comprehensive data on stress management strategies in this study, it was possible to gain important insights into the use of self-threatening coping strategies, proactive coping and emotional coping through positive reframing. Self-endangering behaviors had a detrimental effect on health, while proactive coping showed a health-promoting influence. Positive reframing or the reinterpretation of stressful situations had little to no influence in this study. In order to harness the potential of entrepreneurship for the economy and society, support for the self-employed with regard to their health promotion, as shown in this and similar studies, would be of crucial importance.

The results of the study point to several areas of interest for future research. In addition to testing certain assumptions, such as the mediating effect of proactive coping strategies, an extension of the research model is suggested. It should be noted that the coping construct was not fully captured in this study. Future studies could therefore investigate the stress coping strategies of the self-employed more thoroughly using established measurement instruments. Qualitative study designs could provide a detailed picture of the stress coping strategies of the self-employed.

## Data availability statement

The raw data supporting the conclusions of this article will be made available by the authors, without undue reservation.

## Author contributions

SK: Conceptualization, Formal analysis, Methodology, Visualization, Writing – original draft. SF: Writing – original draft, Writing – review & editing. JS: Resources, Supervision, Writing – review & editing.
